# Association of Social Support With Brain Volume and Cognition

**DOI:** 10.1001/jamanetworkopen.2021.21122

**Published:** 2021-08-16

**Authors:** Joel Salinas, Adrienne O’Donnell, Daniel J. Kojis, Matthew P. Pase, Charles DeCarli, Dorene M. Rentz, Lisa F. Berkman, Alexa Beiser, Sudha Seshadri

**Affiliations:** 1 Center for Cognitive Neurology, Department of Neurology, New York University Grossman School of Medicine, New York; 2 The Framingham Study, Boston, Massachusetts; 3 Harvard Center for Population and Development Studies, Harvard University, Cambridge, Massachusetts; 4 Department of Biostatistics, Boston University School of Public Health, Boston, Massachusetts; 5 Harvard T. H. Chan School of Public Health, Boston, Massachusetts; 6 Turner Institute for Brain and Mental Health, Monash University, Clayton, Victoria, Australia; 7 Department of Neurology, University of California, Davis; 8 Center for Alzheimer Research and Treatment, Department of Neurology, Brigham and Women’s Hospital, Boston, Massachusetts; 9 Department of Neurology, Boston University School of Medicine, Boston, Massachusetts; 10 Glenn Biggs Institute for Alzheimer’s and Neurodegenerative Diseases, University of Texas Health Sciences Center, San Antonio

## Abstract

**Importance:**

Cognitive resilience refers to the general capacity of cognitive processes to be less susceptible to differences in brain structure from age- and disease-related changes. Studies suggest that supportive social networks reduce Alzheimer disease and related disorder (ADRD) risk by enhancing cognitive resilience, but data on specific social support mechanisms are sparse.

**Objective:**

To examine the association of individual forms of social support with a global neuroanatomical measure of early ADRD vulnerability and cognition.

**Design, Setting, and Participants:**

This retrospective cross-sectional analysis used prospectively collected data from Framingham Study participants without dementia, stroke, or other neurological conditions who underwent brain magnetic resonance imaging and neuropsychological testing at the same visit. Data from this large, population-based, longitudinal cohort were collected from June 6, 1997, to December 13, 1999 (original cohort), and from September 11, 1998, to October 26, 2001 (offspring cohort). Data were analyzed from May 22, 2017, to June 1, 2021.

**Exposures:**

Total cerebral volume and, as a modifying exposure variable, self-reported availability of 5 types of social support measured by the Berkman-Syme Social Network Index.

**Main Outcomes and Measures:**

The primary outcome was a global measure of cognitive function. Cognitive resilience was defined as the modification of total cerebral volume’s association with cognition, such that smaller β estimates (presented in SD units) indicate greater cognitive resilience (ie, better cognitive performance than estimated by lower total cerebral volume).

**Results:**

The study included 2171 adults (164 in the original cohort and 2007 in the offspring cohort; mean [SD] age, 63 [10] years; 1183 [54%] female). High listener availability was associated with greater cognitive resilience (β = 0.08, *P* < .001) compared with low listener availability (β = 0.20, *P* = .002). Overall findings persisted after adjustment for potential confounders. Other forms of social support were not significant modifiers (advice: β = −0.04; *P* = .40 for interaction; love-affection: β = −0.07, *P* = .28 for interaction; emotional support: β = −0.02, *P* = .73 for interaction; and sufficient contact: β = −0.08; *P* = .11 for interaction).

**Conclusions and Relevance:**

The results of this cross-sectional cohort study suggest that social support in the form of supportive listening is associated with greater cognitive resilience, independently modifying the association between lower total cerebral volume and poorer cognitive function that would otherwise indicate increased ADRD vulnerability at the preclinical stage. A refined understanding of social support mechanisms has the potential to inform strategies to reduce ADRD risk and enhance cognitive resilience.

## Key Points

**Question:**

What is the association of different forms of social support with an early neuroanatomical marker of Alzheimer disease vulnerability and cognitive function?

**Findings:**

In this cross-sectional study, high (vs low) availability of supportive listening was associated with cognitive resilience, which indicated better global cognitive function than expected for lower cerebral volume. This association was absent for other forms of social support.

**Meaning:**

In psychosocial interventions and related public health strategies to promote neurocognitive health, precise targeting of specific forms of social support, such as supportive listening, may be warranted.

## Teaser

This cross-sectional study uses data from the Framingham Study to examine whether different forms of social support are associated with Alzheimer disease vulnerability and cognitive function.

## Introduction

Studies^1,2^ indicate that not all older adults with substantial neuropathology attributable to Alzheimer disease and related disorders (ADRD) develop dementia. Cognitive resilience is a theoretical concept that attempts to explain this general capacity to remain cognitively unimpaired despite age- or ADRD-related pathological changes,^3,4,5,6^ so clarifying these pathways has important implications for dementia prevention initiatives.

Although broad consensus of operational definitions and research guidelines for cognitive resilience are in development,^7^ a working Alzheimer Association research framework proposes that cognitive resilience–enhancing factors—by definition—modify the association between physical brain changes attributable to age or disease and cognitive performance.^6,8^ The core premise is that cognitive resilience is the difference between an individual’s expected and actual cognitive performance, given their underlying brain structure and level of vulnerability to neuropathological changes. Cognitive resilience is a condition in which an individual has observed cognitive performance better than expected given their brain’s structure. Conversely, low cognitive resilience is a condition in which an individual has cognitive performance that is similar or worse than expected. For the quantification of this otherwise abstract construct, cognitive resilience can be measured directly using an established latent variable modeling approach.^9,10^ This approach quantifies the attenuated correlation of observed global cognitive performance that is better than would be expected for the extent of a neuroanatomical imaging marker of ADRD vulnerability, such as lower cerebral volume on magnetic resonance imaging (MRI).^11,12,13^ Potential cognitive resilience–enhancing factors include educational attainment,^14,15^ physical^16^ and mental^17^ activities, and social relationship measures.^18,19^ Social relationships are of particular interest because an increasing body of evidence suggests factors such as loneliness and social isolation are associated with increased risk of cognitive decline^20,21^ and ADRD pathology.^18,22,23^ Furthermore, a clinicopathological study^18^ of the Rush Memory and Aging Project found that, among 89 dementia-free older adults (mean [SD] age, 84 [6] years), greater social network size at baseline was associated with higher levels of cognitive function before death than would be expected for the extent of neurofibrillary tau tangles and a global measure of ADRD neuropathology found at autopsy (mean [SD] age at death, 87 [6] years). This cognitive resilience was independent of mental and physical activities, depressive symptoms, and chronic medical conditions. Although the measure used in their study^18^ was designed to assess social network size (a structural aspect of social relationships), it is notable that it may resemble measurements of a specific social support domain (functional aspects of social relationships) known as listener availability. Their measure was derived from 3 questions asking about the number of children, family, and friends to whom the respondent feels close, the number with whom they felt at ease and able to talk about private matters or call on for help, and how many of these people they see monthly.^24^ Accurately targeting social relationship factors earlier in life, before the onset of clinical symptoms, may be a promising strategy to reduce ADRD risk and promote neurocognitive health through cognitive resilience pathways.^25^

To better understand underlying cognitive resilience mechanisms potentially associated with social support and to identify targets amenable for intervention trials, a key question to address is whether all supportive functions of social relationships are equally important (eg, availability of supportive listening, advice, love and affection, emotional support, and sufficient contact) or whether a narrower subset of social support domains are responsible for observed associations between composite social support measures and ADRD vulnerability.^26,27^ Thus, we proposed that availability of specific forms of social support enhances cognitive resilience, reducing the clinical expression of lower total cerebral volume as poorer global cognitive function. We focused on total cerebral volume in analyses because (1) neural networks across many cortical and subcortical brain regions support global cognition, (2) proposed preclinical ADRD MRI markers restricted to only a single or subset of regions (similar to neuropsychological markers restricted to only a subset of cognitive domains) might be less sensitive to the broad range of neuropathological mechanisms underlying cognitive decline in a community-based sample,^11,12,13^ and (3) use of total cerebral volume would better represent this heterogeneity in ADRD neuropathogenesis and be informative in generating hypotheses for future studies.^11,28^ We evaluated our hypothesis using one of the largest, longest running, most closely monitored cohorts in the US—the Framingham Study (FS).

## Methods

### Participants

The FS has been described elsewhere.^29^ Briefly, the FS is a community-based study that has enrolled 3 generations of participants. We used the original (n = 5209, enrolled in 1948, biennial examinations) and offspring (n = 5214, enrolled in 1971, quadrennial examinations) cohorts. Offspring participants are children of the original cohort or spouses of original children.^29^ The analytic sample was derived from the 4242 who attended the 25th original examination (June 6, 1997, to December 13, 1999) or seventh offspring examination (September 11, 1998, to October 26, 2001), when social support was assessed. Participants were included if they were 45 years or older, completed the social support assessment, were free of dementia or stroke, and underwent brain MRI and sufficient neuropsychological testing to assess global cognition at the same examination. The data were analyzed from May 22, 2017, to June 1, 2021. Written informed consent was obtained from all participants. All data were deidentified. The institutional review board of Boston University Medical Center approved the consent form and study design. The FS data sets analyzed are available through formal data agreements. Any investigator may access the data through the process outlined at framinghamheartstudy.org. The study followed the Strengthening the Reporting of Observational Studies in Epidemiology (STROBE) reporting guideline.^30^

### Outcome

The primary outcome measure in the study was a global measure of cognitive function that was analyzed using SD units (SDUs). After clinical evaluation, the FS participants underwent standardized neuropsychological test batteries administered by trained research assistants and neuropsychologists. Selected batteries are commonly used in research, have adequate reliability, and cover all major domains assessed in the Alzheimer Disease Center’s Uniform Data Set.^31^ As a measure of global cognitive function, we used a global cognitive score that was developed on a data sample collected at offspring examination 7, with principal component analysis forcing a single component solution. The neuropsychological tasks included in the principal component analysis are the following: Trails Making Test A, Trails Making Test B, Logical Memory (immediate and delayed recall), Visual Reproductions (immediate and delayed recall), Visual Reproductions (immediate and delayed recall), Paired Associate Learning (delayed recall), Hooper Visual Organization Test, and Similarities Test.^32^ The global cognitive score is a weighted sum of standardized scores, where higher scores represent better performance. This method is identical to previous studies^33,34^ and is described in further detail elsewhere^33^; its creation is summarized in eTable 1 in the Supplement.^33,34^

### Cerebral Volume

We used the association of brain structure and cognition to assess cognitive resilience, with smaller β values indicating greater cognitive resilience. We modeled this modification using total cerebral volume from brain MRI as a global neuroanatomical measure of early ADRD vulnerability. Participants underwent scanning with brain-dedicated MRI (1.5 T, Magnetom, Siemens) at the same visit as the neuropsychological assessment. The FS MRI quantification methods have been described, including imaging parameters and sequences, measurement protocols, segmentation methods, reliability, and reproducibility.^35,36,37^ Cerebral volume measures were corrected for head size using the ratio of total brain volume over total cranial volume, multiplied by 100.^35^ Additional details of imaging acquisition and quantification methods are provided in the eMethods in the Supplement.

### Social Supports

Social supports were assessed using the Berkman-Syme Social Network Index (SNI). The SNI is a self-report instrument that measures social network size as well as the type and frequency of social support provided to the respondent; it has been widely used for several decades in longitudinal cohorts derived from the general population, including elderly populations.^38^ Both SNI psychometrics and additional evidence for its validity are available in previous publications.^38^

The SNI has 5 questions that ask participants to select a response that most closely describes their current situation (none of the time, a little of the time, some of the time, most of the time, or all of the time) for the following forms of social support: listening (“Can you count on anyone to listen to you when you need to talk?”), advice (“Is there someone available to give you good advice about a problem?”), love-affection (“Is there someone available to you who shows you love and affection?”), emotional support (“Can you count on anyone to provide you with emotional support?”), and sufficient contact (“Do you have as much contact as you would like with someone you feel close to, someone in whom you can trust and confide?”). Our primary analysis variables are dichotomous indicators of higher level of support (most of or all the time) compared with lower level of support (none, a little, or some of the time). This approach is identical to approaches taken for similar analyses in the FS and other cohorts.^21,26,39^

### Sample Characteristics and Covariates

We parsimoniously assessed sample characteristics and selected covariates a priori to maximize comparability with extant studies.^18,40,41,42^ Covariates included common risk factors for ADRD (age, sex, and educational attainment) as well as age squared (given the nonlinear relationship between age and cerebral volume) and interval (years) from social support assessment to the visit when MRI and neuropsychological measures were both obtained. Depressive symptoms were assessed during the visit SNI was measured using the Center for Epidemiologic Studies–Depression scale with a cutoff score of 16 or higher widely used in the FS and similar cohorts to indicate high depressive symptoms.^43^ Educational attainment was assessed using a 3-level variable (no college degree, some college, or college graduate). Isoelectric focusing of plasma with confirmation by DNA genotype determined apolipoprotein ε4 carrier status.^44^

### Statistical Analysis

Summary statistics were calculated overall and stratified by the age of 65 years. We chose this a priori age cutoff for exploratory stratified analyses within subgroups defined by age given substantially lower ADRD risk for persons younger than 65 years in the FS.^45^ Our analyses focused on the cross-sectional association between total cerebral volume and global cognitive scores, adjusted for age, age squared, sex, educational attainment, and interval between social support measure assessment and the visit when MRI and neuropsychological measures were obtained. Thus, we first regressed each of the total cerebral volume and global cognitive scores onto the primary set of covariates and used the residuals from these regressions (total cerebral volume residual [TCV-r] and global cognitive score residual [GCS-r]) as corresponding exposure and outcome variables, respectively. Associations between social support domains and TCV-r and GCS-r were evaluated using linear regression and reported coefficient estimates (β) in SDUs with 95% CIs and significance test results (*P* values).

To determine the association of social support with cognitive resilience, we examined whether individual social support measures modify the association of brain structure and cognition. For each social support measure, we regressed GCS-r onto TCV-r, social support, and their interaction (social support × TCV-r). We performed this interaction analysis overall and stratified by age group (<65 and ≥65 years of age). Social support measures with significant interactions were identified as modifiers of the association between brain structure and cognition.

We then estimated the association of TCV-r with GCS-r by levels of support (high vs low) for social support measures identified as modifiers. We quantified cognitive resilience as the extent that a high or low level of support modifies TCV-r’s association with GCS-r so smaller β values in the final models—presented as SDUs of global cognition—would indicate greater cognitive resilience (ie, smaller β values reflected reduced clinical expression of lower total cerebral volume as poorer global cognitive function). Conversely, larger β values would represent lower cognitive resilience. This latent variable modeling approach has been commonly used to measure cognitive resilience directly in similar community-based samples.^9,10,46^

To test robustness of results from the chosen categorization of social support measures, we performed sensitivity analyses using social support as a 5-level ordinal variable for each response option. To illustrate the cognitive resilience association, we plotted the estimated linear association between TCV-r and GCS-r by level of social support available. Each social support measure was analyzed in a separate model. To help with the interpretation of our results, we applied a method used in prior work^47,48^ in which we regressed age on global cognitive scores. This method allowed a calculation for SDUs of cognitive decline for each year of aging, thus yielding an interpretation of global cognitive score SDU decrease in terms equivalent to years of cognitive aging. Exploratory interaction analyses used a 2-sided α = .10 to increase sensitivity, identical to a prior FS study^49^ that assessed effect modification. Statistical significance for all other tests was determined using a 2-sided α = .05. All analyses were performed using SAS software, version 9.4 (SAS Institute Inc).

## Results

**Figure 1.**
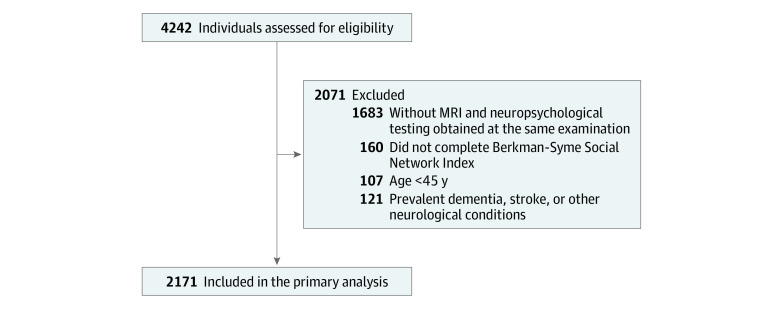
Sample Derivation MRI indicates magnetic resonance imaging.

The study included 2171 adults (164 in the original cohort and 2007 in the offspring cohort; mean [SD] age, 63 [10] years; 1183 [54%] female). (Figure 1 and Table 1). The sample’s characteristics and availability of social support are similar to those observed in the entire cohort and to a report^26^ of social support available in other community-based cohorts. As expected, the distribution of social support scores was skewed; most participants (81%-88%) responded with the highest and second-highest levels of response options available across all 5 domains of social support (eTable 2 in the Supplement). Participants 65 years and older were more likely to have no college degree (407 [45%]), hypertension (566 [63%]), and prevalent cardiovascular disease (196 [22%]). Compared with younger participants, the older participants had lower mean (SD) total cerebral volumes (78.38 [2.05] vs 74.80 [2.51] cm^3^) and global cognitive function scores (0.33 [0.82] vs −0.74 [1.07]) in the younger vs older groups. Age groups did not differ by apolipoprotein ε4 carrier status and depressive symptom burden. The mean (SD) interval from completing the social support measure to the visit when neuropsychological assessment and brain MRI were performed was 0.8 (0.8) years. Examined separately, associations between social support and cerebral volume and between social support and global cognition varied by social support domain (eTable 3 in the Supplement).

**Table 1.** Sample Characteristics^a^

Characteristic	Overall (N = 2171)	Age ≥65 y (n = 898)	Age <65 y (n = 1273)
Cohort			
Original	164 (8)	164 (18)	0 (0)
Offspring	2007 (92)	734 (82)	1273 (100)
Age, mean (SD), y	63 (10)	73 (6)	55 (5)
Sex			
Female	1183 (54)	485 (54)	698 (55)
Male	988 (46)	413 (46)	575 (45)
Educational attainment			
No college degree	723 (33)	407 (45)	316 (25)
Some college	638 (29)	244 (27)	394 (31)
College graduate	810 (37)	247 (28)	563 (44)
Apolipoprotein ε4 carrier status, positive	472 (22)	185 (21)	287 (23)
High depressive symptoms^b^	175 (8)	65 (7)	110 (9)
Stage 1 or higher JNC-VII hypertension	972 (45)	566 (63)	406 (32)
Prevalent cardiovascular disease^c^	265 (12)	196 (22)	69 (5)
Total cerebral volume, mean (SD), cm^3^	76.90 (2.86)	74.80 (2.51)	78.38 (2.05)
Global cognitive function, mean (SD)	−0.11 (1.07)	−0.74 (1.07)	0.33 (0.82)

Abbreviation: JNC, Joint National Committee.

^a^ Data are presented as number (percentage) of patients unless otherwise indicated.

^b^ On the basis of a Center for Epidemiologic Studies–Depression scale score of 16 or higher.

^c^ Includes coronary heart disease, congestive heart failure, peripheral vascular disease, ischemic cardiomyopathy, stroke, and transient ischemic attack.

### Social Support Interactions

We observed an interaction between listener availability and total cerebral volume in identifying global cognition (β = −0.11, *P* = .06 for interaction) (Table 2). This finding indicated that significant differences existed between high and low listener availability with respect to the association between an individual’s global cognitive performance and their underlying total brain volume. Interactions were absent in the other 4 social support domains assessed (advice: β = −0.04; *P* = 0.40 for interaction; love-affection: β = −0.07, *P* = .28 for interaction; emotional support: β = −0.02, *P* = .73 for interaction; and sufficient contact: β = −0.08; *P* = .11 for interaction). The listener availability interaction was present for the participants younger than 65 years (β = −0.16, *P* = .02 for interaction) but not for the participants 65 years and older (β = −0.05, *P* = .61).

**Table 2.** Interactions Between Social Support Domains and Cerebral Volume in Multivariable Models of Global Cognition^a^

Model^b^	Overall (N = 2168)		Age ≥65 y (n = 896)		Age <65 y (n = 1272)	
	β estimate (SE)	*P* value for interaction	β estimate (SE)	*P* value for interaction	β estimate (SE)	*P* value for interaction
Listener × TCV-r	−0.11 (0.06)	.06	−0.05 (0.10)	.61	−0.16 (0.07)	.02
Advice × TCV-r	−0.04 (0.05)	.40	0.02 (0.09)	.83	−0.09 (0.06)	.13
Love-affection × TCV-r	−0.07 (0.06)	.28	−0.06 (0.11)	.59	−0.10 (0.07)	.20
Emotional support × TCV-r	−0.02 (0.06)	.73	−0.01 (0.11)	.93	−0.04 (0.07)	.54
Sufficient contact × TCV-r	−0.08 (0.05)	.11	−0.14 (0.09)	.12	−0.05 (0.06)	.38

Abbreviation: TCV-r, total cerebral volume residual.

^a^ To account for covariates, all models use the residuals of total cerebral volume and global cognitive scores regressed onto the primary set of covariates: age, age squared, sex, educational attainment, and interval from collection of social support measures to time of magnetic resonance imaging and neuropsychological testing. Multivariable regressions modeled global cognitive score residuals as a function of TCV-r, 5 different domains of social support, and the interaction between each social support domain and TCV-r.

^b^ Each type of social support domain was included as a factor in separate models above and as a 2-level variable (high vs low). A high level was defined as responding most of the time or all of the time vs some, little, or none of the time for the respective item: listener: “Can you count on anyone to listen to you when you need to talk?”; advice: “Is there someone available to give you good advice about a problem?”; love-affection: “Is there someone available to you who shows you love and affection?”; emotional support: “Can you count on anyone to provide you with emotional support?”; and sufficient contact: “Do you have as much contact as you would like with someone you feel close to, someone in whom you can trust and confide?”

### Social Support and Cognitive Resilience

**Figure 2.**
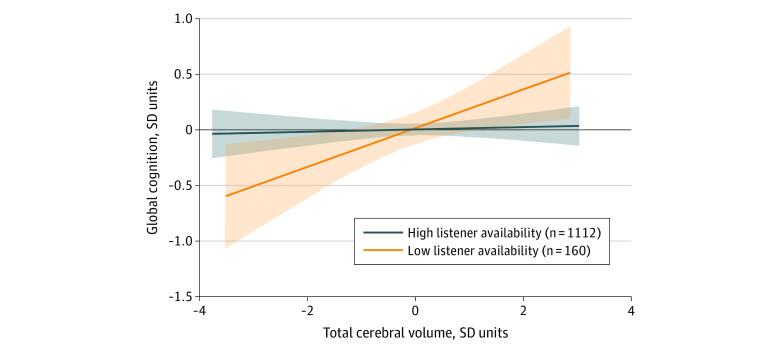
Association Between Cerebral Volume and Global Cognition by Availability of Supportive Listening for Participants 65 Years or Younger To account for covariates, models are based on the residuals of total cerebral volume and global cognitive scores when regressed onto the primary set of covariates: age, age squared, sex, educational attainment, and interval from social support assessment to visit when magnetic resonance imaging and neuropsychological testing were performed. Bands indicate 95% CIs.

High listener availability appeared to modify the association between total cerebral volume and global cognitive score overall (β = 0.08, *P* < .001) (Table 3). This finding was most evident in the younger age group (β = 0.01, *P* = .71). Among participants younger than 65 years with low listener availability, lower brain volume was strongly associated with poorer global cognitive performance (β = 0.17, *P* = .01); for every SDU of decrease in total cerebral volume, cognitive performance decreased by approximately 0.17 SDU (or 4.25 years of cognitive aging). In contrast, among participants with higher listener availability, the same amount of decrease in brain volume was associated with only a 0.01-SDU decrease in cognitive performance (or 0.25 years of cognitive aging). In sensitivity analyses, observations persisted with a more conservative 5-level social support variable (eTable 4 in the Supplement). To help with interpretation of our results visually, we again used exposure and outcome variables as residuals after regressing onto the set of potential confounders and plotted as linear functions identifying the association between TCV-r and GCS-r by level of listener availability. The decrease in global cognition with lower cerebral volumes was more pronounced for participants with low listener availability than for those with high listener availability. This cognitive resilience association was most notable for participants in the younger age group (Figure 2) compared with the overall sample and participants 65 years and older (eFigure 1 and eFigure 2 in the Supplement).

**Table 3.** Multivariable Models of Global Cognition as a Function of Cerebral Volume by Supportive Listener Availability^a^

Listener availability^b^	Overall			Age ≥65 y			Age <65 y		
	No. of participants	Level-specific β estimate (SE)	*P* value	No. of participants	Level-specific β estimate (SE)	*P* value	No. of participants	Level-specific β estimate (SE)	*P* value
High	1898	0.08 (0.02)	<.001	786	0.17 (0.04)	<.001	1112	0.01 (0.03)	.71
Low	270	0.20 (0.06)	.002	110	0.22 (0.11)	.05	160	0.17 (0.07)	.01

^a^ To account for covariates, all models use the residuals of total cerebral volume and global cognitive scores regressed onto the primary set of covariates: age, age squared, sex, educational attainment, and interval from collection of social support measures to time of magnetic resonance imaging and neuropsychological testing. Multivariable regressions modeled global cognitive score residuals as a function of total cerebral volume residuals. Data are presented as β estimate in SD units and SE.

^b^ High listener availability was defined as responding most of the time or all of the time to the item, “Can you count on anyone to listen to you when you need to talk?” Low listener availability was defined as responding with some of the time, little of the time, or none of the time.

## Discussion

This cross-sectional study of 2171 participants not only reaffirmed the neurocognitive benefit of general social support demonstrated by others^18,20,21,22,23^ but also provided additional evidence of a cognitive resilience association between ADRD vulnerability and a specific subtype of social support—supportive listening. The association between the global neuroanatomical measure of early ADRD risk and global cognitive score was reduced in participants with a high level of listener availability compared with those with a low listener availability. When this association is interpreted as cognitive resilience, high listener availability was associated with an increase in cognitive resilience. This association was not observed with other social support domains. The possibility that low listener availability in midlife is an expression of underlying ADRD neuropathology years or decades before clinical diagnosis cannot be ruled out in this observational study.

Our findings are consistent with prior cohort studies^21,26,27,50,51^ that examined associations between social support and lower cognitive function. However, the Atherosclerosis Risk in Communities (ARIC) study^26^ and other studies^27,52^ examined composite social support measures without distinguishing among different subtypes of social support. The ARIC social support measure focused on interpersonal support (combining appraisal, tangible assets, belonging, and self-esteem support subtypes), which was associated with greater global cognition in both Black and White individuals during midlife. Our study extends these findings to clarify that the association was possibly specific to social support involving supportive listening. Another important feature of our study was the additional availability of brain MRI data, permitting us to examine cognitive resilience. Furthermore, these results are consistent with a report^21^ from the FS that cited a 33% lower incident dementia risk among persons who had a listener available to them compared with those who did not (95% CI, 0.49-0.92). Our findings are also consistent with the Rush Memory and Aging Project clinicopathological study^18^ results that supported a correlation between higher social network size—measured using an assessment that resembles the current study’s assessment of listener availability—and cognitive resilience.

Although other forms of social support may relate to ADRD^26,53^ through inflammatory, endocrine, or vascular mechanisms that implicate psychological stress,^54,55,56,57^ supportive listening might uniquely contribute to cognitive resilience through neurobiological mechanisms that diffusely promote experience-induced synaptic plasticity and neurogenesis. For example, supportive listening may be associated with cognitive resilience through pleiotropic neuropeptides more acutely involved in neurobiological processes that play a role in social behavior and executive functioning, such as oxytocin,^58^ or in more chronic mechanisms that involve lifestyle factors^59,60,61^ or neurotrophic factors, such as brain-derived neurotrophic factor, that are critical for synaptogenesis and neural repair and have been linked with both listener availability and lower ADRD risk.^21,62,63^ Furthermore, in a mouse model of ADRD, social interaction has rescued impaired cognitive function through increased brain-derived neurotrophic factor–dependent neurogenesis.^64^ These mechanisms may be associated with the biology of cognitive resilience observed in humans and may partly underlie our finding that listener availability, a specific form of supportive social interaction, was associated with better global cognitive function than would have been expected for lower total cerebral volume.^28,65^

### Strengths and Limitations

This study has important strengths. The FS has obtained a large number of MRIs across the spectrum of brain aging in midlife to late life, in a community-based setting, and with concurrent assessments of cognitive function and multiple social support domains using well-validated instruments for an older adult population. All participants were followed up with standardized protocols, and cognitive outcomes were scored blinded to social support data. Our results address prior gaps in understanding which aspects of social support factors are most strongly associated with cognitive resilience, brain aging, and ADRD.

This study also has limitations. The FS participants are predominantly White adults; however, the overall association of social support with neurocognitive health is likely similar across racially and ethnically diverse cohorts.^26^ Although associations identified cannot establish causality and statistical tests performed may not have been sensitive enough across smaller subgroups, studying associations between social support and cognitive resilience is not readily amenable to randomized clinical trials or feasible for sufficiently large sample sizes to detect small effect sizes; hence, conclusions may rely on observational studies with limited sample sizes in deeply phenotyped cohorts. Our findings are also based on a self-reported assessment of social support availability across 5 domains rather than objective assessment of all supportive social interactions. Although we accounted for many relevant potential confounders, the possibility of unmeasured confounding affecting the overall findings remains. Future studies should further validate our results, investigate temporal dynamics of supportive listening on neurocognitive health, identify candidate neurobiological pathways, and clarify causal mechanisms.

## Conclusions

In this cross-sectional study, high (vs low) availability of supportive listening was associated with cognitive resilience, which was measured directly as better global cognitive function than expected by lower cerebral volume. However, this association was not observed with other types of social support examined. Whether efforts to provide greater access to supportive listeners might delay clinical onset of ADRD remains unknown; however, the results of this study suggest that, when considering supportive psychosocial interventions and other strategies aimed at reducing ADRD risk and promoting neurocognitive health, the precise targeting of specific forms of social support, such as supportive listening, may be warranted.

## Notes

Supplement. **eMethods.** MRI Quantification of Total Cerebral Volume **eTable 1.** Cognitive Tasks and Standardization Used to Create the Global Cognitive Score **eTable 2.** Sample Distribution of Five Social Support Domains, Stratified by Level **eTable 3.** Total Cerebral Volume and Global Cognition Residuals as a Function of Five Social Support Domains **eTable 4.** Multivariable-Models of Global Cognition as a Function of Cerebral Volume and Five Listener Availability Levels **eFigure 1.** Predicted Association Between Cerebral Volume and Global Cognition by Availability of Supportive Listening: All Participants **eFigure 2.** Predicted Association Between Cerebral Volume and Global Cognition by Availability of Supportive Listening: Participants Age ≥65
