# Supplementary material for: Association of Social Support With Brain Volume and Cognition
Source: JAMA Netw Open. 2021 Aug 16;4(8):e2121122. doi: 10.1001/jamanetworkopen.2021.21122 (PMC8369356; doi:10.1001/jamanetworkopen.2021.21122)
Supplement: Supplement. — eMethods. MRI Quantification of Total Cerebral Volume eTable 1. Cognitive Tasks and Standardization Used to Create the Global Cognitive Score eTable 2. Sample Distribution of Five Social Support Domains, Stratified by Level eTable 3. Total Cerebral Volume and Global Cognition Residuals as a Function of Five Social Support Domains eTable 4. Multivariable-Models of Global Cognition as a Function of Cerebral Volume and Five Listener Availability Levels eFigure 1. Predicted Association Between Cerebral Volume and Global Cognition by Availability of Supportive Listening: All Participants eFigure 2. Predicted Association Between Cerebral Volume and Global Cognition by Availability of Supportive Listening: Participants Age ≥65 [file jamanetwopen-e2121122-s001.pdf]

Supplementary Online Content

Salinas J, O'Donnell A, Kojis DJ, et al. Association of social support with brain volume and cognition. *JAMA Netw Open*. 2021;4(8):e2121122. doi:10.1001/jamanetworkopen.2021.21122

eMethods. MRI Quantification of Total Cerebral Volume
eTable 1. Cognitive Tasks and Standardization Used to Create the Global Cognitive Score
eTable 2. Sample Distribution of Five Social Support Domains, Stratified by Level
eTable 3. Total Cerebral Volume and Global Cognition Residuals as a Function of Five Social Support Domains
eTable 4. Multivariable-Models of Global Cognition as a Function of Cerebral Volume and Five Listener Availability Levels
eFigure 1. Predicted Association Between Cerebral Volume and Global Cognition by Availability of Supportive Listening: All Participants
eFigure 2. Predicted Association Between Cerebral Volume and Global Cognition by Availability of Supportive Listening: Participants Age ≥65

This supplementary material has been provided by the authors to give readers additional information about their work.

© 2021 Salinas J et al. *JAMA Network Open.*

eMethods. MRI Quantification of Total Cerebral Volume

Acquisition Parameters

Imaging was performed with a Siemens Magnetom 1.0 Tesla field strength magnetic resonance machine using a double spin-echo coronal imaging sequence of 4 millimeter contiguous slices from nasion to occiput with repetition time of 2,420 msec, echo time (TE) of TE1 20/ TE2 90 msec; echo train length 8 msec; field of view 22 cm and a 182 × 256 acquisition matrix interpolated to a 256 × 256 with one excitation. Approximately 90% of scans were performed in Massachusetts; the remainder were performed out-of-state and used a 1.5T machine with identical scan protocol. Off-site scanners had test scans with verification that they were performed correctly according to the Framingham Study MRI scan protocol.

Image Analysis

MRI scan digital information was transferred after acquisition to the central laboratory directed by a co-author (CD) for processing and analysis. Analyses were all conducted blind to participant identifying information. Images were evaluated with semiautomatic segmentation analyses using operator-guided removal of non-brain elements by operator-guided tracing of the dura matter within the cranial vault. This included the middle cranial fossa and was above the posterior fossa and cerebellum. The cranial vault measure derived was defined as total cranial volume and was used as a head size estimate to correct for established sex differences in head size.

Total cerebral brain volume was quantified using a multi-step process starting with image segmentation to define brain matter from cerebral spinal fluid (CSF). Subtraction of the second echo image from the first echo image yielded a difference image. Following image segmentation into brain matter and CSF, the operator returned to the image to measure lobar brain volumes. To preserve measurement precision, segmented brain-CSF images were rotated separately from the original image.

The image was transformed into anatomic standard space; the operator then returned to the image and identified brain lobar and regional CSF measures. Volumes were all calculated as the sum of the pixels within the identified region of interest multiplied by the pixel volume in milliliters. Repeat analysis of intra- and inter-rater reliabilities were consistently above 0.90.[36] The measured total cerebral volume was corrected for head size using the ratio of total cerebral volume over total cranial volume, multiplied by 100.[34]

© 2021 Salinas J et al. *JAMA Network Open.*

eTable 1. Cognitive Tasks and Standardization Used to Create the Global Cognitive Score[a]

Cognitive Task	Natural Log-Transformations	Standardizing Formula[b]	Component Loading[c]
Trails Making Test A	-log(score)	(score – 0.68) / 0.34	0.13
Trails Making Test B	-log(score)	(score + 0.22) / 0.45	0.18
Trails Making Test (B – A)	-log(2 + score)	(score + 1.03) / 0.22	0.16
Logical Memory – Immediate Recall		(score – 11.55) / 3.4	0.14
Logical Memory – Delayed Recall		(score – 10.61) / 3.59	0.15
Visual Reproductions – Immediate Recall		(score – 9.07) / 3.15	0.17
Visual Reproductions – Delayed Recall		(score – 8.2) / 3.36	0.18
Paired Associate Learning – Delayed Recall		(score – 8.3) / 1.46	0.13
Hooper Visual Organization Test	-log(31 – score)	(score + 1.65) / 0.52	0.14
Similarities Test		(score – 16.77) / 3.55	0.14

[a] Adapted from Pase et al. 2016.[33]

[b] Natural log transformed cognitive tasks were used to create the standardized variables where applicable.

[c] Global cognitive score calculated by summing the products of the standardizing formulas and the component loadings for each cognitive task.

© 2021 Salinas J et al. *JAMA Network Open.*

eTable 2. Sample Distribution of Five Social Support Domains, Stratified By Level

**Social Support Domain[a] (n=2171)**	**No. of Participants (%)**
Listener	
None of the time	10 (1%)
Little of the time	79 (4%)
Some of the time	181 (8%)
Most of the time	748 (35%)
All of the time	1150 (53%)
Advice	
None of the time	26 (1%)
Little of the time	99 (5%)
Some of the time	289 (13%)
Most of the time	850 (39%)
All of the time	901 (42%)
Love-Affection	
None of the time	23 (1%)
Little of the time	98 (5%)
Some of the time	146 (7%)
Most of the time	502 (23%)
All of the time	1394 (65%)
Emotional support	
None of the time	20 (1%)
Little of the time	87 (4%)
Some of the time	184 (9%)
Most of the time	700 (32%)
All of the time	1175 (54%)
Sufficient contact	
None of the time	29 (1%)
Little of the time	112 (5%)
Some of the time	244 (11%)
Most of the time	725 (34%)
All of the time	1056 (49%)

[a] For each type of social support, participants responded to the following items on the Berkman-Syme Social Network Index: listening, “Can you count on anyone to listen to you when you need to talk?”; advice, “Is there someone available to give you good advice about a problem?”; love-affection, “Is there someone available to you who shows you love and affection?”; emotional support, “Can you count on anyone to provide you with emotional support?”; and, sufficient contact, “Do you have as much contact as you would like with someone you feel close to, someone in whom you can trust and confide?”.

© 2021 Salinas J et al. *JAMA Network Open.*

eTable 3. Total Cerebral Volume and Global Cognition Residuals as a Function of Five Social Support Domains[a]

	TCV-r		GCS-r	
**Models[b]**	**Beta Estimate (SE)**	***P* Value**	**Beta Estimate (SE)**	***P* Value**
Overall (n=2171)				
Listener	0.14 (0.03)	.03	0.16 (0.06)	.02
Advice	0.17 (0.05)	.002	0.05 (0.05)	.40
Love-Affection	0.09 (0.07)	.19	0.06 (0.07)	.37
Emotional support	0.11 (0.06)	.09	0.04 (0.06)	.56
Sufficient contact	0.11 (0.06)	.04	0.05 (0.06)	.35
Age <65 (n=1273)				
Listener	0.14 (0.08)	.07	0.01 (0.08)	.91
Advice	0.11 (0.07)	.12	-0.06 (0.06)	.35
Love-Affection	0.10 (0.08)	.19	0.09 (0.07)	.24
Emotional support	0.09 (0.08)	.28	0.01 (0.07)	.88
Sufficient contact	0.10 (0.07)	.15	0.03 (0.06)	.65
Age ≥65 (n=898)				
Listener	0.14 (0.11)	.18	0.37 (0.12)	.001
Advice	0.25 (0.09)	.005	0.20 (0.10)	.04
Love-Affection	0.06 (0.12)	.60	0.01 (0.12)	.93
Emotional support	0.14 (0.10)	.19	0.07 (0.11)	.51
Sufficient contact	0.15 (0.10)	.13	0.10 (0.11)	.37

Abbreviations: TCV-r, total cerebral volume residual; GCS-r, global cognitive score residual.

[a] To account for covariates, all models use the residuals of total cerebral volume and global cognitive scores regressed onto the primary set of covariates: age, age^2, sex, education, and time interval from collection of social support measures to time of MRI and neuropsychological testing. Multivariable regressions separately modeled TCV-r and GCS-r as a function of five different domains of social support.

[b] Each type of social support domain was included as a predictor in separate models above and as a two-level variable, high versus low. A high level was defined as responding "most of the time or all of the time" versus "some, little, or none of the time" for the respective item:
Listener, "Can you count on anyone to listen to you when you need to talk?"
Advice, "Is there someone available to give you good advice about a problem?"
Love-affection, "Is there someone available to you who shows you love and affection?"
Emotional support, "Can you count on anyone to provide you with emotional support?"
Sufficient contact, "Do you have as much contact as you would like with someone you feel close to, someone in whom you can trust and confide?"

© 2021 Salinas J et al. *JAMA Network Open.*

eTable 4. Multivariable Models of Global Cognition as a Function of Cerebral Volume and Five Listener Availability Levels[a]

Level of Supportive Listener Availability[b]	No. of Participants (overall)	Level-Specific Beta Estimate (SE)	*P* Value
None of the time	10	-0.23 (0.30)	.48
Little of the time	79	0.20 (0.11)	.07
Some of the time	181	0.21 (0.08)	.006
Most of the time	748	0.09 (0.04)	.02
All of the time	1150	0.08 (0.03)	.006

[a] To account for covariates, all models use the residuals of total cerebral volume and global cognitive scores regressed onto the primary set of covariates: age, age^2, sex, education, and time interval from collection of social support measures to time of MRI and neuropsychological testing. Multivariable regressions modeled global cognitive score residuals as a function of total cerebral volume residuals. Data are presented as beta estimate in standard deviation units and standard error (SE).

[b] Response to the item, "Can you count on anyone to listen to you when you need to talk?"

© 2021 Salinas J et al. *JAMA Network Open.*

eFigure 1. Predicted Association Between Cerebral Volume and Global Cognition By Availability of Supportive Listening: All Participants

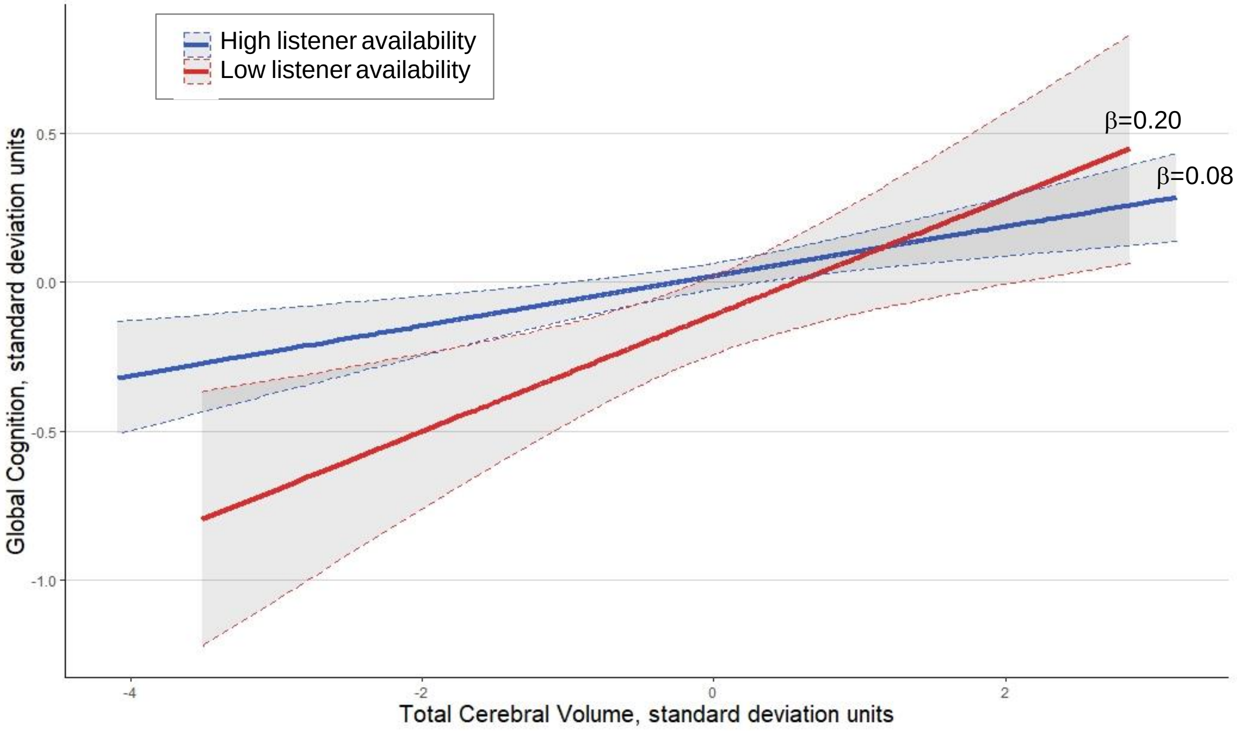

To account for covariates, models are based on the residuals of total cerebral volume and global cognitive scores when regressed onto the primary set of covariates: age, age^2, sex, education, and time interval from social support assessment to visit when MRI and neuropsychological testing were obtained. Blue line=High listener availability (n=1898). Red line=Low listener availability (n=279). Bands indicate 95% confidence intervals.

© 2021 Salinas J et al. *JAMA Network Open.*

eFigure 2. Predicted Association Between Cerebral Volume and Global Cognition By Availability of Supportive Listening: Participants Age ≥65

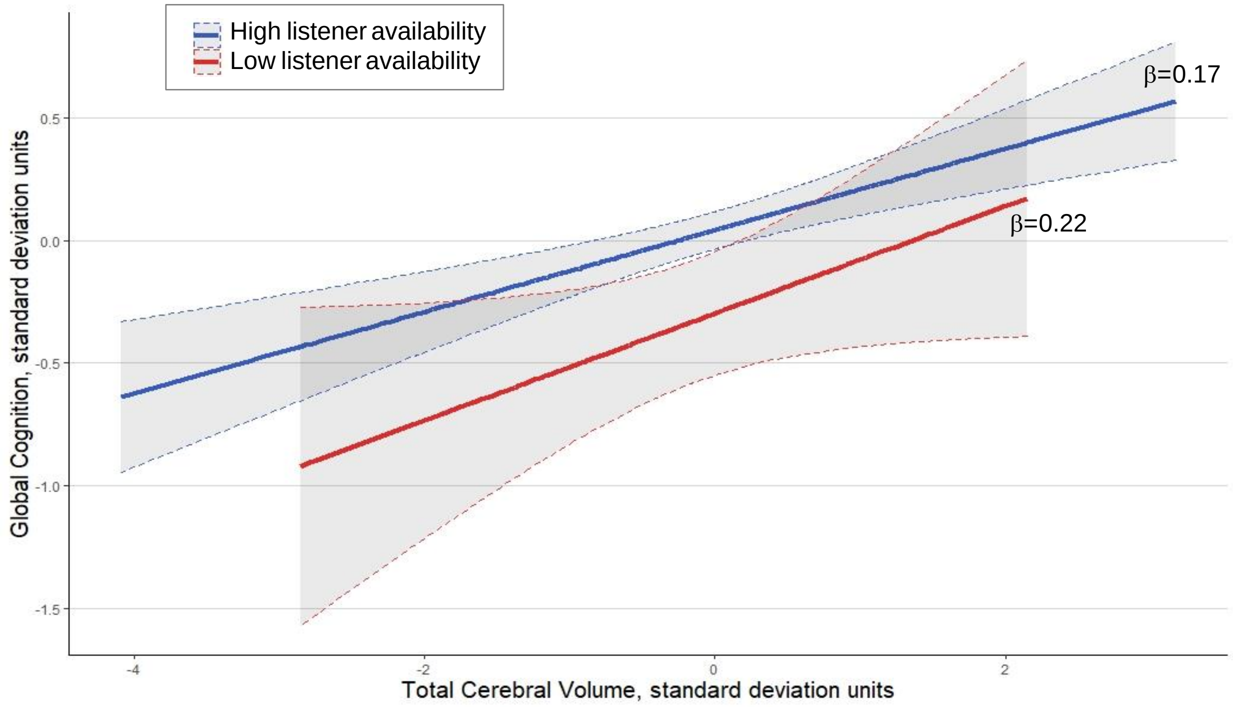

To account for covariates, models are based on the residuals of total cerebral volume and global cognitive scores when regressed onto the primary set of covariates: age, age^2, sex, education, and time interval from social support assessment to visit when MRI and neuropsychological testing were obtained. Blue line=High listener availability (n=786). Red line=Low listener availability (n=110). Bands indicate 95% confidence intervals.

© 2021 Salinas J et al. *JAMA Network Open.*

References

1. Bennett DA, Wilson RS, Boyle PA, Buchman AS, Schneider JA. Relation of neuropathology to cognition in persons without cognitive impairment. *Ann Neurol.* 2012;72(4):599-609.
2. Beker N, Ganz A, Hulsman M, et al. Association of Cognitive Function Trajectories in Centenarians With Postmortem Neuropathology, Physical Health, and Other Risk Factors for Cognitive Decline. *JAMA Netw Open.* 2021;4(1):e2031654.
3. Stern Y, Barnes CA, Grady C, Jones RN, Raz N. Brain reserve, cognitive reserve, compensation, and maintenance: operationalization, validity, and mechanisms of cognitive resilience. *Neurobiol Aging.* 2019;83:124-129.
4. Barulli D, Stern Y. Efficiency, capacity, compensation, maintenance, plasticity: emerging concepts in cognitive reserve. *Trends Cogn Sci.* 2013;17(10):502-509.
5. Stern Y. Cognitive reserve in ageing and Alzheimer's disease. *Lancet Neurol.* 2012;11(11):1006-1012.
6. Stern Y, Arenaza-Urquijo EM, Bartres-Faz D, et al. Whitepaper: Defining and investigating cognitive reserve, brain reserve, and brain maintenance. *Alzheimers Dement.* 2018.
7. Stern Y, Albert M, Barnes CA, Cabeza R, Pascual-Leone A, Rapp P. Collaboratory on research definitions for reserve and resilience in cognitive aging and dementia. https://reserveandresilience.com/. Published 2021. Accessed February 15, 2021.
8. Bartrés-Faz D, Arenaza-Urquijo E, Ewers M, et al. Theoretical frameworks and approaches used within the Reserve, Resilience and Protective Factors professional interest area of the Alzheimer's Association International Society to Advance Alzheimer's Research and Treatment. *Alzheimer's & Dementia: Diagnosis, Assessment & Disease Monitoring.* 2020;12(1):e12115.
9. Reed BR, Mungas D, Farias ST, et al. Measuring cognitive reserve based on the decomposition of episodic memory variance. *Brain.* 2010;133(Pt 8):2196-2209.
10. Zahodne LB, Manly JJ, Brickman AM, Siedlecki KL, Decarli C, Stern Y. Quantifying cognitive reserve in older adults by decomposing episodic memory variance: replication and extension. *J Int Neuropsychol Soc.* 2013;19(8):854-862.
11. Whitwell JL, Tosakulwong N, Weigand SD, et al. Does amyloid deposition produce a specific atrophic signature in cognitively normal subjects? *Neuroimage Clin.* 2013;2:249-257.
12. Carmichael O, Mungas D, Beckett L, et al. MRI predictors of cognitive change in a diverse and carefully characterized elderly population. *Neurobiol Aging.* 2012;33(1):83-95.
13. Farias ST, Mungas D, Reed B, et al. Maximal brain size remains an important predictor of cognition in old age, independent of current brain pathology. *Neurobiol Aging.* 2012;33(8):1758-1768.
14. Bennett DA, Wilson RS, Schneider JA, et al. Education modifies the relation of AD pathology to level of cognitive function in older persons. *Neurology.* 2003;60(12):1909-1915.
15. Mungas D, Gavett B, Fletcher E, Farias ST, DeCarli C, Reed B. Education amplifies brain atrophy effect on cognitive decline: implications for cognitive reserve. *Neurobiol Aging.* 2018;68:142-150.
16. Okonkwo OC, Schultz SA, Oh JM, et al. Physical activity attenuates age-related biomarker alterations in preclinical AD. *Neurology.* 2014;83(19):1753-1760.
17. Valenzuela MJ, Sachdev P. Brain reserve and dementia: a systematic review. *Psychol Med.* 2006;36(4):441-454.
18. Bennett DA, Schneider JA, Tang Y, Arnold SE, Wilson RS. The effect of social networks on the relation between Alzheimer's disease pathology and level of cognitive function in old people: a longitudinal cohort study. *Lancet Neurol.* 2006;5(5):406-412.
19. Flatt JD, Rosso AL, Aizenstein HJ, et al. Social Network Size and Cranial Magnetic Resonance Imaging Findings in Older Adults: The Cardiovascular Health Study. *J Am Geriatr Soc.* 2015;63(11):2430-2432.
20. Donovan NJ, Wu Q, Rentz DM, Sperling RA, Marshall GA, Glymour MM. Loneliness, depression and cognitive function in older US adults. *Int J Geriatr Psychiatry.* 2017;32(5):564-573.
21. Salinas J, Beiser A, Himali JJ, et al. Associations between social relationship measures, serum brain-derived neurotrophic factor, and risk of stroke and dementia. *Alzheimers Dement (N Y).* 2017;3(2):229-237.
22. Donovan NJ, Okereke OI, Vannini P, et al. Association of Higher Cortical Amyloid Burden With Loneliness in Cognitively Normal Older Adults. *JAMA Psychiatry.* 2016;73(12):1230-1237.
23. d'Oleire Uquillas F, Jacobs HIL, Biddle KD, et al. Regional tau pathology and loneliness in cognitively normal older adults. *Transl Psychiatry.* 2018;8(1):282.
24. Barnes LL, Mendes de Leon CF, Wilson RS, Bienias JL, Evans DA. Social resources and cognitive decline in a population of older African Americans and whites. *Neurology.* 2004;63(12):2322-2326.

© 2021 Salinas J et al. *JAMA Network Open.*

25. Shah H, Albanese E, Duggan C, et al. Research priorities to reduce the global burden of dementia by 2025. *The Lancet Neurology.* 2016;15(12):1285-1294.
26. Kats D, Patel MD, Palta P, et al. Social support and cognition in a community-based cohort: the Atherosclerosis Risk in Communities (ARIC) study. *Age Ageing.* 2016;45(4):475-480.
27. Seeman TE, Lusignolo TM, Albert M, Berkman L. Social relationships, social support, and patterns of cognitive aging in healthy, high-functioning older adults: MacArthur studies of successful aging. *Health Psychol.* 2001;20(4):243-255.
28. Jack CR, Jr., Bennett DA, Blennow K, et al. NIA-AA Research Framework: Toward a biological definition of Alzheimer's disease. *Alzheimers Dement.* 2018;14(4):535-562.
29. Kannel WB, Feinleib M, McNamara PM, Garrison RJ, Castelli WP. An investigation of coronary heart disease in families. The Framingham offspring study. *Am J Epidemiol.* 1979;110(3):281-290.
30. Weintraub S, Salmon D, Mercaldo N, et al. The Alzheimer's Disease Centers' Uniform Data Set (UDS): the neuropsychologic test battery. *Alzheimer Dis Assoc Disord.* 2009;23(2):91-101.
31. Wechsler D. WAIS-R : Wechsler adult intelligence scale-revised. In: New York, N.Y. : Psychological Corporation, [1981] ©1981; 1981.
32. Davies G, Armstrong N, Bis JC, et al. Genetic contributions to variation in general cognitive function: a meta-analysis of genome-wide association studies in the CHARGE consortium (N=53949). *Mol Psychiatry.* 2015;20(2):183-192.
33. Pase MP, Beiser A, Enserro D, et al. Association of Ideal Cardiovascular Health With Vascular Brain Injury and Incident Dementia. *Stroke.* 2016;47(5):1201-1206.
34. Albert M, Massaro J, DeCarli C, et al. Profiles by sex of brain MRI and cognitive function in the framingham offspring study. *Alzheimer Dis Assoc Disord.* 2010;24(2):190-193.
35. Fletcher E, Singh B, Harvey D, Carmichael O, DeCarli C. Adaptive image segmentation for robust measurement of longitudinal brain tissue change. *Annu Int Conf IEEE Eng Med Biol Soc.* 2012;2012:5319-5322.
36. DeCarli C, Massaro J, Harvey D, et al. Measures of brain morphology and infarction in the framingham heart study: establishing what is normal. *Neurobiol Aging.* 2005;26(4):491-510.
37. Berkman LF, Syme SL. Social networks, host resistance, and mortality: a nine-year follow-up study of Alameda County residents. *Am J Epidemiol.* 1979;109(2):186-204.
38. Salinas J, Ray RM, Nassir R, et al. Factors Associated With New-Onset Depression Following Ischemic Stroke: The Women's Health Initiative. *J Am Heart Assoc.* 2017;6(2).
39. Andel R, Vigen C, Mack WJ, Clark LJ, Gatz M. The effect of education and occupational complexity on rate of cognitive decline in Alzheimer's patients. *J Int Neuropsychol Soc.* 2006;12(1):147-152.
40. Rabin JS, Schultz AP, Hedden T, et al. Interactive Associations of Vascular Risk and beta-Amyloid Burden With Cognitive Decline in Clinically Normal Elderly Individuals: Findings From the Harvard Aging Brain Study. *JAMA Neurol.* 2018.
41. Pase MP, Beiser A, Aparicio H, et al. Interarm differences in systolic blood pressure and the risk of dementia and subclinical brain injury. *Alzheimers Dement.* 2016;12(4):438-445.
42. Radloff LS. The CES-D scale a self-report depression scale for research in the general population. *Appl Psychol Meas.* 1977;1(3):385-401.
43. Welty FK, Lahoz C, Tucker KL, Ordovas JM, Wilson PW, Schaefer EJ. Frequency of ApoB and ApoE gene mutations as causes of hypobetalipoproteinemia in the Framingham Offspring population. *Arterioscler Thromb Vasc Biol.* 1998;18(11):1745-1751.
44. Chene G, Beiser A, Au R, et al. Gender and incidence of dementia in the Framingham Heart Study from mid-adult life. *Alzheimers Dement.* 2015;11(3):310-320.
45. Lamballais S, Zijlmans JL, Vernooij MW, Ikram MK, Luik AI, Ikram MA. The Risk of Dementia in Relation to Cognitive and Brain Reserve. *J Alzheimers Dis.* 2020;77(2):607-618.
46. Baril AA, Beiser AS, Mysliwiec V, et al. Slow-Wave Sleep and MRI Markers of Brain Aging in a Community-Based Sample. *Neurology.* 2021;96(10):e1462-e1469.
47. Tsao CW, Himali JJ, Beiser AS, et al. Association of arterial stiffness with progression of subclinical brain and cognitive disease. *Neurology.* 2016;86(7):619-626.
48. Nishtala A, Piers RJ, Himali JJ, et al. Atrial fibrillation and cognitive decline in the Framingham Heart Study. *Heart rhythm : the official journal of the Heart Rhythm Society.* 2018;15(2):166-172.
49. von Elm E, Altman DG, Egger M, et al. The Strengthening the Reporting of Observational Studies in Epidemiology (STROBE) statement: guidelines for reporting observational studies. *Ann Intern Med.* 2007;147(8):573-577.

© 2021 Salinas J et al. *JAMA Network Open.*

50. Kuiper JS, Zuidersma M, Oude Voshaar RC, et al. Social relationships and risk of dementia: A systematic review and meta-analysis of longitudinal cohort studies. *Ageing Res Rev.* 2015;22:39-57.
51. Ertel KA, Glymour MM, Berkman LF. Effects of social integration on preserving memory function in a nationally representative US elderly population. *Am J Public Health.* 2008;98(7):1215-1220.
52. Akbaraly TN, Portet F, Fustinoni S, et al. Leisure activities and the risk of dementia in the elderly: results from the Three-City Study. *Neurology.* 2009;73(11):854-861.
53. Rentz DM, Mormino EC, Papp KV, Betensky RA, Sperling RA, Johnson KA. Cognitive resilience in clinical and preclinical Alzheimer's disease: the Association of Amyloid and Tau Burden on cognitive performance. *Brain Imaging Behav.* 2017;11(2):383-390.
54. Ihle A, Rimmele U, Oris M, Maurer J, Kliegel M. The Longitudinal Relationship of Perceived Stress Predicting Subsequent Decline in Executive Functioning in Old Age Is Attenuated in Individuals with Greater Cognitive Reserve. *Gerontology.* 2020;66(1):65-73.
55. Non AL, Rimm EB, Kawachi I, Rewak MA, Kubzansky LD. The effects of stress at work and at home on inflammation and endothelial dysfunction. *PloS one.* 2014;9(4):e94474.
56. Yang YC, McClintock MK, Kozloski M, Li T. Social isolation and adult mortality: the role of chronic inflammation and sex differences. *J Health Soc Behav.* 2013;54(2):183-203.
57. Yang YC, Li T, Frenk SM. Social network ties and inflammation in U.S. adults with cancer. *Biodemography Soc Biol.* 2014;60(1):21-37.
58. Owen SF, Tuncdemir SN, Bader PL, Tirko NN, Fishell G, Tsien RW. Oxytocin enhances hippocampal spike transmission by modulating fast-spiking interneurons. *Nature.* 2013;500(7463):458-462.
59. Pettigrew C, Soldan A, Zhu Y, et al. Cognitive reserve and rate of change in Alzheimer's and cerebrovascular disease biomarkers among cognitively normal individuals. *Neurobiol Aging.* 2020;88:33-41.
60. Christakis NA, Fowler JH. The collective dynamics of smoking in a large social network. *N Engl J Med.* 2008;358(21):2249-2258.
61. Christakis NA, Fowler JH. The spread of obesity in a large social network over 32 years. *N Engl J Med.* 2007;357(4):370-379.
62. Weinstein G, Beiser AS, Choi SH, et al. Serum brain-derived neurotrophic factor and the risk for dementia: the Framingham Heart Study. *JAMA Neurol.* 2014;71(1):55-61.
63. Taylor WD, Züchner S, McQuoid DR, Steffens DC, Blazer DG, Krishnan KR. Social support in older individuals: the role of the BDNF Val66Met polymorphism. *Am J Med Genet B Neuropsychiatr Genet.* 2008;147B(7):1205-1212.
64. Hsiao YH, Hung HC, Chen SH, Gean PW. Social interaction rescues memory deficit in an animal model of Alzheimer's disease by increasing BDNF-dependent hippocampal neurogenesis. *J Neurosci.* 2014;34(49):16207-16219.
65. Kaur B, Himali JJ, Seshadri S, et al. Association between neuropathology and brain volume in the Framingham Heart Study. *Alzheimer Dis Assoc Disord.* 2014;28(3):219-225.

© 2021 Salinas J et al. *JAMA Network Open.*
